# Cancer cells can be killed mechanically or with combinations of cytoskeletal inhibitors

**DOI:** 10.3389/fphar.2022.955595

**Published:** 2022-10-10

**Authors:** Ajay Tijore, Bo Yang, Michael Sheetz

**Affiliations:** ^1^ Centre for Biosystems Science and Engineering, Indian Institute of Science, Bangalore, India; ^2^ Mechanobiology Institute, National University of Singapore, Singapore, Singapore; ^3^ Department of Biochemistry and Molecular Biology, University of Texas Medical Branch, Galveston, TX, United States

**Keywords:** mechanobiology, cancer, mechanosensitivity, transformed growth, cytoskeleton, ultrasound, apoptosis

## Abstract

For over two centuries, clinicians have hypothesized that cancer developed preferentially at the sites of repeated damage, indicating that cancer is basically “continued healing.” Tumor cells can develop over time into other more malignant types in different environments. Interestingly, indefinite growth correlates with the depletion of a modular, early rigidity sensor, whereas restoring these sensors in tumor cells blocks tumor growth on soft surfaces and metastases. Importantly, normal and tumor cells from many different tissues exhibit transformed growth without the early rigidity sensor. When sensors are restored in tumor cells by replenishing depleted mechanosensory proteins that are often cytoskeletal, cells revert to normal rigidity-dependent growth. Surprisingly, transformed growth cells are sensitive to mechanical stretching or ultrasound which will cause apoptosis of transformed growth cells (Mechanoptosis). Mechanoptosis is driven by calcium entry through mechanosensitive Piezo1 channels that activate a calcium-induced calpain response commonly found in tumor cells. Since tumor cells from many different tissues are in a transformed growth state that is, characterized by increased growth, an altered cytoskeleton and mechanoptosis, it is possible to inhibit growth of many different tumors by mechanical activity and potentially by cytoskeletal inhibitors.

## Introduction

Tumors are well known for their heterogeneity (Dagogo-Jack and Shaw, 2018). This phenomenon generally occurs both within tumors (intra-tumor) or between tumors (inter-tumor) and thus makes it difficult to find common features of different tumor types that would enable common treatments. In early studies of tumor cells, they grew on soft agar, while normal cells required rigid surfaces to proliferate (Hamburger and Salmon, 1977). As a property of many if not most tumor cells, growth on soft agar or “transformed growth” can be a feature that could potentially be exploited in treating different tumor types. For example, recent studies show that transformed growth correlates with mechanically induced apoptosis (mechanoptosis). Mechanical forces from stretch, shear, or ultrasound can induce tumor cell mechanoptosis (Regmi et al., 2017; Tijore et al., 2020; Singh et al., 2021; Tijore et al., 2021). Thus, in the context of physiological relevance, mechanoptosis could explain the benefits of exercise for cancer patients with a wide diversity of cancers (Wang and Zhou, 2021). Although the basis of mechanoptosis is still not fully understood, it is a common feature of tumor cells that can possibly be exploited in treatments of cancers.

### Tumor cells show altered rigidity sensing due to absence of rigidity sensors

An important question is whether or not growth on soft agar represents a phenotypic change of the state of tumor cells that correlates with mechanoptosis. From a number of studies, it is interesting that the loss of early rigidity sensing correlates with growth on soft agar (Raval et al., 2003; Bharadwaj et al., 2005; Wolfenson et al., 2016; Yang et al., 2020). It is important to note that substrate rigidity will affect a variety of different cell pathways particularly over longer periods (Paszek et al., 2005; Doss et al., 2020) and that early rigidity sensing refers to a specific sensing complex. The early rigidity sensor has been defined as a sarcomeric unit of about 2 μm in length that has a myosin bipolar filament in the middle with antiparallel actin filaments anchored to integrins and the extracellular matrix through alpha actinin (Meacci et al., 2016; Wolfenson et al., 2016; Saxena et al., 2017a). During early cell spreading on fibronectin, the sarcomeric unit assembles rapidly and contracts matrix a total of about 100 nm before relaxing (total time 40–60 s) (Lohner et al., 2019). If the force at the peak displacement exceeds 25 pN, then the surface is considered rigid; and if less than 25 pN, the surface is soft and anoikis through DAPK1 is initiated (Qin et al., 2018a). In the cases of tumor cells early rigidity sensing is lost since they are deficient in one or more mechanosensory cytoskeletal protein(s) that are part of the early rigidity sensor, restoration of normal levels of the missing protein(s) typically results in rigidity-dependent growth (Yang et al., 2020). Conversely, in normal cells when single mechanosensory cytoskeletal proteins of the rigidity sensor are depleted, cells exhibit rigidity-independent transformed growth on soft agar. These behaviors have proven true for a number of tumor and normal cells across different tissues (Yang et al., 2020; Tijore et al., 2021). For example, in a screen of 36 ovarian tumor cell lines ranging from epithelial to mesenchymal, they all appear to lack early rigidity sensing, with most cell lines missing known components of the rigidity sensor (half of the cell lines are depleted in tropomyosin 2.1) (Simpson et al., 2022).

Since many of the proteins that are part of the early rigidity sensor are cytoskeletal proteins, it is logical to ask if their depletion or replenishment alters other protein levels in the cells or only involves the proteins in question. Interestingly, RNAseq comparisons of the cells with versus those without the cytoskeletal protein, tropomyosin 2.1 (Tpm2.1), show that the levels of 700–1000 mRNAs are significantly altered (Yang et al., 2020). Thus, it seems that the loss of the early rigidity sensor causes a major change in cell composition that is, indicative of a change in phenotype. Another question is how the changes in cytoskeletal properties result from the change in the cell state. Depletion of cytoskeletal proteins such as tropomyosin 2.1, Filamin A and myosin IIA can result in major changes in the organization of the cytoskeleton throughout the cytoplasm and not just in cortical regions (Luo et al., 2013). Although relatively less is known about how this portion of the cytoskeleton influences the organization of cytoplasmic organelles, it is clear that the transformed cell state involves changes in the ER-mitochondrial stress pathway that could increase the mechanoptosis of the cells (Doghman-Bouguerra and Lalli, 2019; Kim et al., 2020).

### Tumor cells are sensitive to mechanoptosis and killed by mechanical forces

Over the last few decades, numerous studies have focused on understanding the role of biochemical cues in tumor development and progression. However, in recent years, it has been documented that tumor growth and progression can be influenced by external mechanical stresses (Yuan et al., 2016; Emon et al., 2018; Riehl et al., 2021). Recent literature has suggested that tumor cells are vulnerable to damage by external mechanical stresses. For instance, physiologically relevant shear forces were found to kill adherent tumor cells *via* an apoptotic pathway triggered by bone morphogenetic protein receptor, Smad1/5 and p38 MAPK (Lien et al., 2013). Another study observed that fluid shear stresses sensitize cancer cells to TRAIL-induced apoptosis via caspase activation (Mitchell and King, 2013). Similarly, circulating tumor cells were killed at high shear forces *via* an oxidative stress-induced mitochondrial apoptotic pathway (Regmi et al., 2017). Further, it was reported that exercise or mechanical stretching of tumors caused tumor regression in a mouse model (Betof et al., 2015; Berrueta et al., 2018). All these studies indicated that mechanical stresses could inhibit cancer growth.

**FIGURE 1:**
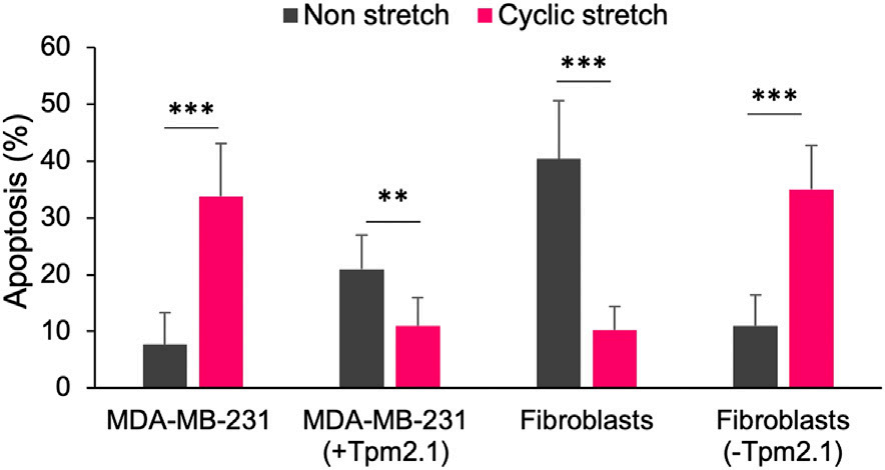
Bar graph showing the percentage of apoptotic cells with or without 24 h of 5% mechanical stretch for MDA-MB-231 breast cancer cells with or without expressed tropomyosin 2.1 (Tpm2,1) and mouse embryo fibroblasts before or after Tpm2.1 knockdown. Adapted from (Tijore et al., 2021).

If we look at these findings in a broader context, they might be relevant since physically active muscle tissue shows a low risk of tumor formation. In fact, muscle-associated tumors are rare and don’t even make it to the list of 36 commonly occurring tumors worldwide (Bray et al., 2018). In support of these facts, several clinical studies have found that there is a strong correlation between exercise and tumor growth inhibition. For instance, recent studies reported that resistance exercise, a form of exercise to improve muscle tone and endurance was associated with a 33% lower risk of all causes of mortality in cancer survivors (Hardee et al., 2014). Another excellent review article summarized the positive impact of exercise on cancer mortality, recurrence and treatment-related side-effects (Cormie et al., 2017). In fact, an article from the NCI reported that 13 cancer types including breast cancer were inhibited by exercise (Moore et al., 2016). Although there is no exact explanation of the basis for the correlation between exercise and tumor growth inhibition, there is a definite possibility that periodic mechanical stresses generated during exercise cause selective tumor cell killing. In further support of the idea that tumor cells are sensitive to exercise-induced strains, we found tumor cell mechanoptosis was caused by 5% strains as shown in Figure 1 (Tijore et al., 2021) and such strains are easily attained with normal exercise. Both tumor cells and normal cells depleted of early rigidity sensors showed the same behavior. Again, the tissue of cell origin did not appear to affect the mechanoptosis. Thus, the phenotypic changes with transformed growth include increased mechanoptosis.

### Mechanical forces cause intracellular calcium uptake to induce mitochondria-mediated apoptosis

**FIGURE 2:**
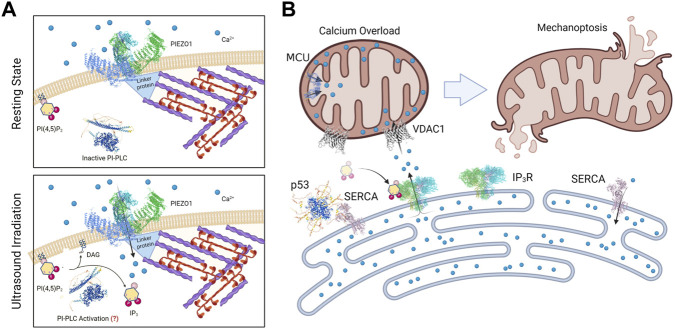
Diagrams of a working model for Mechanoptosis of transformed growth cells. In **(A)** the Piezo1 channel is shown in complex with cytoplasmic proteins that are linked to an actomyosin network that needs to be active for mechanoptosis. In the lower panel the hydrolysis of PIP2 is shown to produce IP3 although the mechanism of activation of phosphatidyl inositol phospholipase C (PI-PLC) is not understood nor is the link to Piezo1 activity. In **(B)** the rise in IP3 level activates the release of ER calcium by the IP3 receptor that is linked to the VDAC channel, causing a rise in mitochondrial calcium levels leading to apoptosis. The ER calcium pump, SERCA is shown in complex with p53 at these sites.

In terms of the mechanism of mechanically-induced tumor cell apoptosis, there is a logical pathway that involves the susceptibility of tumor cells to damage from activation of an ER-mitochondrial stress pathway. In both the cases of stretch- and ultrasound-activated apoptosis, an early step is dependent on mechanosensitive Piezo1 channels. Since tumor cells are sensitive to phospholipase C activation (Halder et al., 2014), calcium entry through Piezo1 or other mechanochannels at the plasma membrane could activate phospholipase C hydrolysis of PIP2 to release inositol triphosphate, IP3; however, the direct activation of IP3 release by Piezo1 action has not been seen, which implicates a secondary mechanism in transformed growth cells (Kuriyama et al., 2022). Binding of IP3 to the IP3 receptor in the ER causes calcium loading of mitochondria through the VDAC1 channel in the mitochondria that is, physically linked to IP3R (Doghman-Bouguerra and Lalli, 2019; Simoes et al., 2020). Many anticancer compounds cause apoptosis through calpain activation as a result of an increase in intracellular calcium downstream of PLC dependent IP3 release (Wu et al., 2010; Lu et al., 2012; Huang et al., 2014; Cho et al., 2015; Kerkhofs et al., 2018; Bae et al., 2021). This is similar to other systems where Piezo1 activation is linked to apoptosis through mitochondrial malfunction in the ER-mitochondrial stress pathway (Hope et al., 2019). It is important to note that Piezo1 is implicated in many mechanical cell processes through a variety of different models (see reviews (De Felice and Alaimo, 2020; Dombroski et al., 2021)). The particular factors that activate mechanoptosis including the prolonged periodic mechanical stimulation make it somewhat different than those other models for Piezo1 functions. What has struck us is that many different cell lines in the transformed growth state regardless of origin and EMT state are sensitive to mechanoptosis. All these findings are summarized in Figure 2 and the ER-mitochondrial stress model comes from recent reviews (Simoes et al., 2020; Kerkhofs et al., 2018; Doghman-Bouguerra and Lalli, 2019).

### Genes associated with ER-mitochondrial stress pathway are upregulated in the transformed cell state

The detailed analyses of changes in cellular mRNA composition (RNAseq) with rigidity-independent growth versus rigidity-dependent growth indicate that a number of genes associated with the ER-mitochondria stress pathway are upregulated in the transformed growth state (Yang et al., 2020). Upon comparison of which genes are increased in the transformed growth state of both human foreskin fibroblasts without Tpm2.1 (HFF) and MDA-MB-231 tumor cells that normally lack Tpm2.1, there are 86 genes in common (Table 1). When MDA-MB-231 cells exhibit rigidity-dependent growth upon tropomyosin 2.1 expression, there are 13 upregulated genes that are also upregulated in normal HFFs (Table 1). In recent papers, a number of common genes that are highly expressed in the transformed growth state are featured and several of them are involved with mitochondrial stress and ROS (green highlighting), cancer functions (blue highlighting) as well as mitochondrial DNA repair (orange highlighting) (Doghman-Bouguerra and Lalli, 2019; Simoes et al., 2020; Wu et al., 2021). Perhaps most surprising is that thirteen of the identified proteins are interferon inducible (IFI and OAS proteins) and many are involved in anti-viral activities. Since these pathways are commonly activated in tumor cells and are upregulated during the transformed growth of fibroblasts, it is logical to consider them as possible contributors to the mechanoptosis sensitivity of the transformed growth state.

**TABLE 1:** RNAseq data showing the thirteen genes that are elevated in expression (co-expressed) in normal human foreskin fibroblasts (HFF) and MDAMB-231 cells expressing Tpm2.1. In the HFF cells after Tpm2.1 knockdown and the MDA-MB-231 cells, there were 86 genes that were elevated in expression (inversely co-expressed).

Co-expressed (*n* = 13)	Inverserly Co-expressed (*n* = 86)				
CDCA7	APOBEC3G	CYP1B1	IF127	NCOA7	SAMD9L
CDK19	APOL1	DDX58	IF144	OAS1	SECTM1
CREB3L4	ASPHD2	DDX60	IF144L	OAS2	SLC22A23
FAM102B	ATF3	DDX6OL	IFI6	OAS3	SSTR2
GJA1	BAMBI	DHX58	IFIH1	OASL	STX11
ITGA10	BATF2	EPSTI1	IFIT1	PARP12	THEMIS2
KISS1	CCL5	FAM46A	IFIT2	PARP14	TMEM140
MARCKSL1	CD24	GBP4	IFIT3	PATL2	TMEM229B
NTM	CDK18	GBP5	IFITM10	PCDH1	TNIFSF10
PTPRQ	CEACAM1	GCH1	L23A	PIK3AP1	TRANK1
RGCC	CFB	GIMAP2	IL4I1	PLEKHA4	TYMP
TMEM191C	CH25H	GNAO1	ISG15	PLEKHF1	USP18
TPM2	CMPK2	GRIP2	ISG20	PRRG4	ZNF467
	CPEB3	HERC5	KIF26B	RARRES3	ZNFX1
	CTSS	HERC6	LAMP3	RRAD	
	CXCL10	HLA-B	LMO2	RSAD2	
	CXCL11	HLA-F	MX1	RTP4	
	CXCL16	IDO1	MX2	SAMD9	

Based upon common changes in gene levels in both fibroblasts and breast tumor cells with transformed growth, it is logical to argue that the genes involved are responsible for changes in cytoskeletal organization of tumor cells. For example, transformed cells produce high traction forces on matrices (Indra et al., 2011; Alcoser et al., 2015) (Yang et al., 2020), whereas the cytoplasm of transformed cells is softer which helps them to efficiently metastasize to different tissues (Lv et al., 2021). The organization of cell cytoplasm involves a cohesive actin network that can bridge between adhesions (Cai et al., 2010; Rossier et al., 2010). Further, nodes in the network are throughout cytoplasm and can consequently contribute to the stiffness of the cell upon indentation (Luo et al., 2013; Luo et al., 2016). What is currently unclear is which proteins are involved in the changes in cytoskeletal organization of tumor cells as well as how those changes might be related to changes in ER-mitochondrial organization that would enable mechanoptosis.

### *In vitro* testing using combinations of cytoskeletal drugs

Combination therapy, the use of two or multiple drugs at the same time for treatment has become one of the fastest growing therapeutic areas for cancer treatment (Mokhtari et al., 2021). On the other hand, cytoskeleton targeting drugs, especially drugs targeting microtubules and actin filaments, are used as chemotherapeutic drugs in the clinics (Kubiak et al., 2020). However, many of the approved cytoskeletal drugs, for example, Paclitaxel (Taxol), mainly work through preventing mitosis possibly by stabilizing microtubules (Gallego-Jara et al., 2020). Other cytoskeleton components that are part of the rigidity sensing modules and cell growth are still largely overlooked.

Our recent findings have defined a number of components of the rigidity sensors including DAPK1, PTPN12, AXL, EGFR, Calpain 2, Src, and the cytoskeletal proteins such as myosin IIA, tropomyosin 2.1, alpha actinin, filamin A, tropomodulin, and α_v_β_3_ integrin (Meacci et al., 2016; Wolfenson et al., 2016; Yang et al., 2016; Saxena et al., 2017a; Saxena et al., 2017b; Qin et al., 2018b) (Ghassemi et al., 2012). Of these components, DAPK1, PTPN12, AXL, EGFR, tropomyosin 2.1 and alpha actinin have roles as tumor suppressors in some cases. In the case of DAPK1, it localizes with the rigidity sensors at the sites of integrin-ECM attachment. On soft pillars, it is activated by PTPN12 phosphatase and rapidly leaves the pillars to potentially activate apoptosis if enough adhesions are soft (Qin et al., 2018b). On stiff pillars, its density increases along with other adhesion proteins potentially due to greater tyrosine kinase activity. Although we expect that a similar set of events takes place in transformed cells at adhesions, the details have not been worked out. Once we understand the important changes in the transformed growth state that enable unregulated growth, it would be possible to consider targeted drugs as well as mechanical perturbations to inhibit tumor cell growth.

Thus, these findings raise a question of whether or not cytoskeleton drugs which targeting rigidity sensing components, should be part of cancer combination therapy. The challenges include not only selecting the best combination of drugs but also deciding the best concentration of each drug. Fortunately, with the help of Artificial Intelligence (AI) and high throughput preclinical screening systems, researchers can effectively design the combination therapy for different diseases or even individual patients in a remarkably short time (Rashid et al., 2018). It is logical to screen for different combination of drugs that will inhibit transformed cell growth but not normal cell growth. Altogether, the combination of various cytoskeleton drugs along with checkpoint inhibitors, chemotherapeutic drugs, or mechanical therapies could serve as a new avenue for cancer treatment in the future.

### Tumor cell mechanoptosis: Mechanism and future therapeutic directions

What are the requirements for tumor cell mechanoptosis? The model in Figure 2 highlights the fact that myosin contractility is needed for ultrasound-induced mechanoptosis (Singh et al., 2021). This is not easy to interpret, since myosin inhibition dramatically decreases actin polymerization and causes the loss of the cytoplasmic actin networks that provide cytoplasmic coherence (Cai et al., 2010; Rossier et al., 2010; Luo et al., 2013). Many conditions including soft matrices cause reduced myosin contractility in normal and transformed growth cells and could thereby decrease mechanoptosis. Alternatively, there are many drugs that activate tumor cell apoptosis through a similar ER-mitochondrial stress pathway to the one that is, activated by mechanical forces and may be synergistic with mechanoptosis (Kumar et al., 2012; Lin et al., 2014; Lu et al., 2019).

When tumor cells are able to grow for long periods, tumor cells can differentiate to better grow in their microenvironment. For example, propagating tumors in mice and serially selecting tumor cells from specific tissues established tumor lines that target specific tissues (Kang et al., 2003). When the growth of those tumor cells was tested on matrices of different stiffness, better growth was found on surfaces that mimicked the stiffness of the tissues that they targeted (Kostic et al., 2009). The differentiation potential of tumor cells indicates that it will be difficult to find a common property of tumor cells. Further, there are studies that show phenotypic changes of tumor cells with different matrix environments (Paszek et al., 2005) or with modifications of integrin function (Weaver et al., 1997).

Looking back to the basis of the transformed growth state and the relation to regeneration and inflammation (Sheetz, 2019), transformed growth could be stimulated by normal inflammatory processes. The increased expression of miR-21 downregulates Tpm2.1 expression and causes the loss of early rigidity sensing, which occurs in many tumor cells (An et al., 2018; Yan et al., 2018; Wang et al., 2019). Thus, there are many factors like TGF-ß that cause an increase in miR21 expression, which will promote transformed growth (Qian et al., 2009; Dai et al., 2017; Despotovic et al., 2021). These conditions are reversible and can be related to the microenvironment as well as circulating growth factors related to inflammation. For these reasons, tumor growth can be episodic and respond to general trauma or acute inflammation but will be slowed in non-inflammatory conditions with depletion of miR-21. In this example, tumor growth is still related to the loss of rigidity sensing and the transformed growth state, which means that treatments targeting the transformed growth state may still be effective.

## Conclusion

As we have noted, the transformed growth state is a general property of tumor cells irrespective of tissue origin and thus, a treatment that targets the transformed state can be effective against different tumor types. Characterization of tumor cells shows that they have altered cytoskeleton functions and can undergo mechanoptosis. In early cancer studies, tumor cells were described as being in a transformed growth state and the transformed growth state has been shown recently to depend upon depletion of the early rigidity sensor module that is, activated in normal cells as they spread on matrices (Wolfenson et al., 2016; Yang et al., 2020). Normal cells can assume the transformed state upon depletion of single cytoskeletal proteins involved in the rigidity sensing module and conversely tumor cells will assume a rigidity-dependent state upon restoration of normal levels of the depleted proteins that then enable rigidity sensing (Yang et al., 2020). The most common protein that is depleted in many cancers is tropomyosin 2.1 and this can be the effect of miR21 causing depletion of the tropomyosin 2.1 mRNA because miR21 is upregulated in cancer development, wound repair and inflammation (Qian et al., 2009; Dai et al., 2017; An et al., 2018). An important point is that the transformed state is common to most tumor cells; therefore, treatments that target the transformed state might be effective against many tumor types.

Although we don’t fully understand the molecular pathways that account for the differences between the transformed and normal growth states, there are some aspects of cytoskeletal organization that could be involved. For example, the actin cytoskeleton of normal cells involves myosin contraction as well as actin crosslinking by filamin A and the depletion of either of these proteins will cause cell transformation (Cai et al., 2010; Luo et al., 2013; Luo et al., 2016; Wolfenson et al., 2019). Thus, it is logical to suggest that the actin filament networks in tumor cells are altered as compared to the normal cells. In the future, additional studies are required to further understand the differences between normal and transformed cell cytoskeletons and how they might be exploited to develop tumor treatments.
